# Toll-Like Receptor -1, -2, and -6 Polymorphisms and Pulmonary Tuberculosis Susceptibility: A Systematic Review and Meta-Analysis

**DOI:** 10.1371/journal.pone.0063357

**Published:** 2013-05-14

**Authors:** Yuxiang Zhang, Tingting Jiang, Xiuyun Yang, Yun Xue, Chong Wang, Jiyan Liu, Xing Zhang, Zhongliang Chen, Mengyuan Zhao, Ji-Cheng Li

**Affiliations:** 1 Institute of Cell Biology, Zhejiang University, Hangzhou, Zhejiang, China; 2 Department of Pharmacology, University of Pennsylvania, Philadelphia, Pennsylvania, United States of America; 3 Department of Respiratory Medicine, Tongde Hospital of Zhejiang, Hangzhou, Zhejiang, China; 4 Henan University of Science and Technology, Luoyang, Henan, China; Hopital Raymond Poincare - Universite Versailles St. Quentin, France

## Abstract

**Background:**

A large number of studies have investigated whether polymorphisms in the Toll-like receptor (TLR) genes are implicated in susceptibility to tuberculosis (TB) in different populations. However, the results are inconsistent and inconclusive.

**Methods:**

A literature search was conducted using the PubMed, EMBASE, Medline (Ovid), ISI Web of Knowledge and Chinese National Knowledge Infrastructure (CNKI). A meta-analysis on the associations between the *TLR1* G1805T, *TLR2* T597C, T1350C, G2258A, and *TLR6* C745T polymorphisms and TB risk was carried out by comparison using different genetic models.

**Results:**

In total, 16 studies from 14 articles were included in this review. In meta-analysis, significant associations were observed between the *TLR2* 2258AA (AA vs. AG+AG, OR 5.82, 95% CI 1.30–26.16, *P* = 0.02) and TLR6 745TT (TT vs. CT+CC, OR 0.61, 95% CI 0.39–0.97, *P = *0.04) polymorphisms and TB risk. In the subgroup analysis by ethnicity, Africans and American Hispanic subjects with the *TLR1* 1805T allele had an increased susceptibility, whereas Asian and European subjects with the *TLR2* 2258A allele had an increased susceptibility to TB.

**Conclusions:**

The meta-analysis indicated that *TLR2* G2258A is associated with increased TB risk, especially in Asians and Europeans. *TLR1* G1805T is associated with increased TB in Africans and American Hispanics. *TLR6* C745T is associated with decreased TB risk. Our systematic review and meta-analysis reported an interesting preliminary conclusion, but this must be validated by future large-scale and functional studies in different populations.

## Introduction

Tuberculosis (TB) is a contagious and potentially fatal disease caused by various strains of mycobacteria, usually Mycobacterium tuberculosis (Mtb) in humans. It can infect almost any part of the body, but manifests mainly as an infection of the lungs and kills more people each year than any other single infectious disease. It is estimated by WHO that about one-third of the current global population is infected asymptomatically with Mtb, of whom 5–10% will develop clinical disease during their lifetime [1]. This fact together with other substantial evidence indicates that variations in host hereditary factors play an important role in susceptibility to TB [2]. The susceptibility to Mycobacterium can result from either inadequate or excessive acute inflammation. Thus, single nucleotide polymorphisms (SNPs) in genes that control the balance of pro- and anti- inflammatory response could confer different TB risk to the host, as exemplified in zebra fish and human studies [3]. Host genotype-specific therapies can optimize the inflammatory response to Mycobacterial infections [4]. This personalized therapy requires the knowledge of the host immune response against Mtb and association of host genotypes with TB risk.

**Figure 1:**
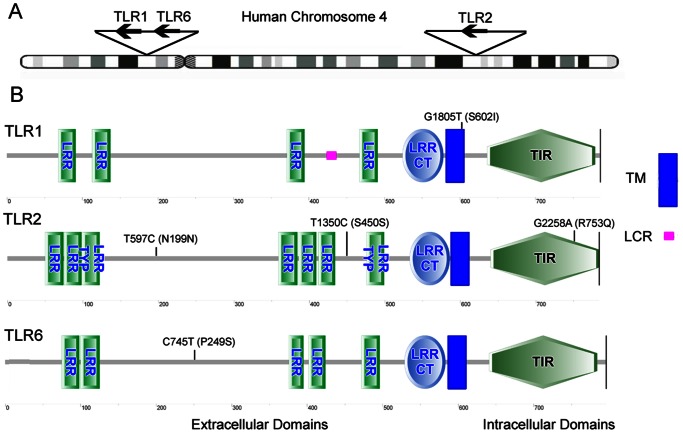
Schematic representation of the TLR-1, -2 and -6 genes, proteins and selected sequence variants. (A) Schematic representation of human chromosome 4 showing location of TLR-1, -2 and -6 genes. (B) Schematic representation of the five selected variants in TLR proteins. *TLR1* G1805T is in the TM domain; TLR2 T597, T1350 and *TLR6* C745T are in extracellular domains between the LRRs. *TLR2* G2258A is in the intracellular TIR domain. LRR: Leucine-rich repeats; LRR TYP: LRR Typical Subfamily; LRR CT: C-terminal LRR Domain; TIR: Toll-Interleukin-1 Receptor Domain; TM: Trans-membrane Region; LCR: Low Complexity Region. Different domains are detected by SMART.

Toll-like receptors (TLRs) can recognize pathogen-associated molecular patterns (PAMPs) of Mtb and initiate signaling pathways that lead to the activation of the innate immune response, cytokines and formation of the adaptive immune response [5]. The major receptors for the Mtb are TLRs-1, -2, -4, -6 and -9 [6]–[10], among them TLR2 and TLR4 play a key role in the reorganization process. TLR2 can form heterodimers with either TLR1 or TLR6 to sense the PAMPs of Mtb and activate macrophages and dendritic cells through adaptor proteins MyD88 and TIRAP [11]. On the other hand, TLR4, together with CD14 and MD2, could recognize the lipopolysaccharide (LPS) and initiate signal transduction in the MyD88-dependent pathway. It can also function via the MyD88-independent pathway involving TRIF-dependent type 1 interferon response [11]. Many studies have reported that polymorphisms in these TLR-2 and -4 pathways can regulate inflammatory response to bacterial components and thus could impact innate immune response and clinical susceptibility to TB [12]–[14]. However, two recent meta-analyses have studied the TB susceptibility and *TLR4* Asp299Gly, Thr399Ile, and *TIRAP* Ser180Leu polymorphisms, which are the most studied SNPs in these two genes [15], [16]. Their results revealed that these polymorphisms were unlikely to substantially contribute to TB susceptibility and raised the question whether the polymorphisms in TLR pathways are associated with the TB risk. Nevertheless, numerous studies have also been performed to investigate other polymorphisms in *TLR* genes and their association with TB. Among them, *TLR1* G1805T, *TLR2* T597C, T1350C, G2258A, *TLR6* C745T are most studied as potential risk factors for TB. Figure 1 is the schematic representation of these polymorphisms, which showed that *TLR2* T597C, T1350C and *TLR6* C745T are in the extracellular domains, *TLR1* G1850T is in the trans-membrane (TM) domain and *TLR2* G2258A is in the Toll-Interleukin-1 receptor (TIR) domain.

Many studies have reported the association of these polymorphisms with TB; however, the results are inconsistent and inconclusive due to limited sample sizes and different study populations. Therefore, in this article, we chose these polymorphisms as our candidates and performed a systematic review and meta-analysis, based on literature identification until 1^st^ January 2013 to summarize the associations between these polymorphisms and TB susceptibility.

## Materials and Methods

### Selection of Studies for Analysis

**Figure 2:**
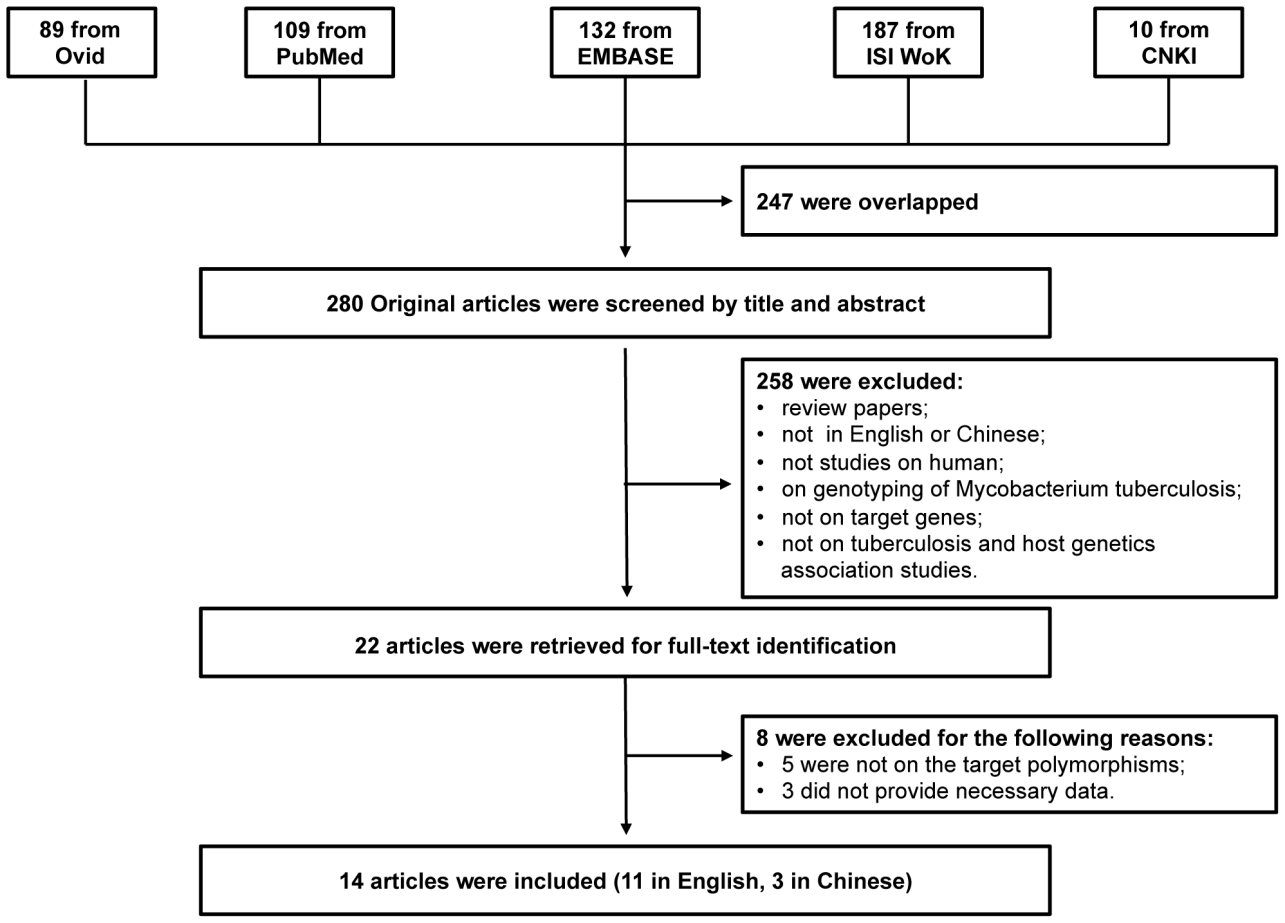
Flow chart showing the study selection procedure. Ovid: Ovid MEDLINE; ISI WoK: ISI Web of Knowledge; CNKI: China National Knowledge Infrastructure.

We searched the PubMed, EMBASE, Medline (Ovid), ISI Web of Knowledge and Chinese National Knowledge Infrastructure (CNKI) to identify studies of the association between TB susceptibility and TLRs polymorphisms until 1^st^ January 2013. The key words were: ‘Mycobacterium tuberculosis’ or ‘tuberculosis’ in combination with ‘polymorphism’ or ‘variant’ or ‘genotype’ or ‘allele’ or ‘mutation’, and in combination with ‘toll’ or ‘TLR’ or ‘Toll-like receptor’ or ‘Toll like receptor’. The search results were limited to English and Chinese language articles. All retrieved titles and abstracts were examined for relevant studies on the association between TB and TLR gene polymorphisms. Studies were selected if they met the following criteria: 1) case-control studies of unrelated individuals; 2) evaluation of polymorphisms *TLR1* G1805T, *TLR2* T597C, T1350C, G2258A, *TLR6* C745T and TB susceptibility; 3) genotype distribution in both cases and controls were available. Exclusion criteria were: 1) study design based on family or sibling pairs; 2) genotype frequencies not reported; and 3) data from reviews and abstracts. Additional studies were also identified by hand searching reference lists of original studies and review articles including meta-analysis. The PRISMA Checklist in Table S1 was used as a guide to format this article and the systematic review process was described in the flowchart of Figure 2. The schematic of the SNPs in these genes in Figure 1 are generated based on the protein annotation from SMART database [17].

### Data Extraction

For all studies, we extracted the following data from original publications: first author and year of publication; distribution of genotypes for each polymorphism among cases and controls; characteristics of the study design and the study population (study base, numbers and mean age of cases and controls, TB diagnosis, HIV status, source of controls, matching criteria and host ethnicity).

### Statistical Analyses

Hardy-Weinberg Equilibrium (HWE) was examined in controls by asymptotic Pearson’s Chi-square test for each polymorphism in each study. The association between polymorphism and TB was estimated by means of odds ratios (OR) and corresponding 95% confidence intervals (CI) comparing cases to controls. Between-study heterogeneity was tested by the Q test and I^2^ test [18], and the heterogeneity was considered significant if *P*-value was less than 0.05. Fixed-effects models were adopted when *P*-value is more than 0.05; otherwise random-effects models were used. The funnel plot, Begg’s test and Egger’s test were used to evaluate the publication bias [19], [20]. Statistical analyses were carried out using the Stata/SE 11.0 (College Station, TX, USA) and Review Manager 5.0 software (Oxford, England).

## Results

### Characteristics of Included Studies

A total of 527 articles were achieved by literature search. As shown in Figure 2, after excluding those overlapped between the databases, 280 abstracts were retrieved for detailed evaluation. Twenty-two studies addressing the association of targeted-gene polymorphisms and TB were identified. Full-text article retrieval excluded 8 of them and the exclusion criteria were shown in Table S2. Finally, 16 studies from 14 articles, 11 in English [14], [21]–[30] and 3 in Chinese [31]–[33] were included in this review and meta-analysis. Among them, there were six studies for *TLR1* G1805T, seven studies for *TLR2* T597C, six studies for *TLR2* T1350C, eleven studies for *TLR2* G2258A, and four studies for *TLR6* C745T. As shown in Table 1, the study participants were from diverse descents including African, Asian, European and American. The pooled sample size was 7373 (3757 cases and 3616 controls). The genotype and allele distributions of all the polymorphisms are shown in Table 2. In three studies, the genotype distributions in controls were deviated from HWE [24], [25].

**Table 1:** Characteristics of the 16 studies included in the meta-analysis.

Author	Year	Country	Ethnicity	Age, years Mean ± SD or Mean (Range)		Samplesn		Genotyping method
				Cases	Controls	Cases	Controls	
Che, et al.	2010	China	Chinese Han	/	/	115	156	PCR-Sequencing
Chen, et al.	2010	China	Taiwanese	56.7±18.7	53.9±11.5	184	184	PCR-Sequencing
Dalgic, et al.	2011	Turkey	Turkish	8.11±4.89	8.52±4.55	124	200	PCR-RFLP
Etokebe, et al.	2010	Croatia	Croatian Caucasian	51.03±18.71	41.84±11.90	186	551	PCR-Sequencing
Jin, et al.	2007	China	Chinese Han	26–63	19–55	170	199	PCR-SSP
Ma, et al.	2010	China	Chinese Han	34.75±16.67	38.17±17.39	543	544	PCR-RFLP
Ma, et al. _a	2007	United States	African American	/	/	339	194	PCR-Sequencing
Ma, et al. _b	2007	United States	European American	/	/	180	110	PCR-Sequencing
Ma, et al. _c	2007	United States	Hispanic American	/	/	375	114	PCR-Sequencing
Ogus, et al.	2004	Turkey	Turkish	35.4±13.5	35.9±14.8	151	116	PCR-SSP
Salvaraj, et al.	2010	India	Dravidian	34.92±11.42	32.33±9.75	206	212	PCR-RFLP
Sanchez, et al.	2010	Colombia	Colombian	39 (26–51)	42 (25–54)	499	320	PCR-MS
Thuong, et al.	2007	Vietnam	Vietnamese	/	/	358	389	PCR-MS
Uciechowski, et al.	2011	Germany	German	58.5±16.8	30.5±7.7	45	49	PCR-Sequencing
Xue, et al.	2010	China	Chinese Han	39.5±17.9	26.3±8.5	205	203	PCR-Sequencing
Yu	2008	China	Chinese Han	/	/	77	75	PCR-RFLP

PCR = polymerase chain reaction; SSP = sequence-specific primers; RFLP = restriction fragment length polymorphism; MS = mass spectroscopy.

**Table 2:** Genotype and allele distribution of TLR -1, -2, and -6 polymorphisms in TB and controls.

SNP	Study	Case					Control					HWE	
		GG	GT	TT	G	T	GG	GT	TT	G	T	X^2^	*P*
*TLR1*	Ma, et al.	510	32	1	1052	34	509	34	1	1052	36	0.294	0.588
G1805T	Ma, et al. _a	4	63	272	71	607	13	61	120	87	301	1.795	0.180
(rs5743618)	Ma, et al. _b	107	61	12	275	85	63	33	14	159	61	6.956	0.008
	Ma, et al. _c	20	83	272	123	627	14	39	61	67	161	3.518	0.061
	Salvaraj et al.	1	9	192	11	393	0	16	189	16	394	0.338	0.561
	Uciechowski et al.	36	5	4	77	13	24	19	6	67	31	0.525	0.469
		TT	TC	CC	T	C	TT	TC	CC	T	C	X^2^	*P*
*TLR2*	Thuong et al.	177	138	35	492	208	205	154	18	564	190	2.633	0.105
T597C	Etokebe et al.	66	84	40	216	164	162	244	83	568	410	0.298	0.585
(rs3804099)	Ma, et al. _a	46	165	128	257	421	29	100	65	158	230	0.889	0.346
	Ma, et al. _b	55	90	35	200	160	41	47	22	129	91	1.562	0.211
	Ma, et al. _c	133	191	51	457	293	18	80	16	116	112	18.601	0.000
	Sanchez, et al.	173	220	72	566	364	95	153	52	343	257	0.514	0.473
	Che, et al.	52	54	9	158	72	68	68	20	204	108	0.214	0.644
		TT	TC	CC	T	C	TT	TC	CC	T	C	X^2^	*P*
*TLR2*	Che, et al.	60	48	7	168	62	79	61	16	219	93	0.670	0.413
T1350C	Chen, et al.	131	45	8	307	61	121	55	8	297	71	0.297	0.586
(rs3804100)	Etokebe, et al.	159	26	1	344	28	483	67	1	1033	69	0.709	0.400
	Ma, et al. _a	299	38	2	636	42	169	25	0	363	25	0.920	0.337
	Ma, et al. _b	151	24	5	326	34	101	9	0	211	9	0.200	0.655
	Ma, et al. _c	312	62	1	686	64	100	14	0	214	14	0.488	0.485
		GG	GA	AA	G	A	GG	GA	AA	G	A	X^2^	*P*
*TLR2*	Ogus, et al.	124	13	14	261	41	107	7	2	221	11	12.783	0.000
G2258A	Ma, et al. _a	337	2	0	676	2	194	0	0	388	0	/	/
(rs5743708)	Ma, et al. _b	171	9	0	351	9	105	5	0	215	5	0.059	0.807
	Ma, et al. _c	374	1	0	749	1	110	4	0	224	4	0.036	0.849
	Jin, et al.	99	71	0	269	71	168	31	0	367	31	1.420	0.233
	Yu	76	1	0	153	1	75	0	0	150	0	/	/
	Salvaraj, et al	192	1	0	385	1	198	1	0	397	1	0.001	0.972
	Sanchez, et al.	463	3	0	929	3	296	4	0	596	4	0.014	0.907
	Xue, et al.	204	1	0	409	1	202	1	0	405	1	0.001	0.972
	Ma, et al.	543	0	0	1086	0	544	0	0	1088	0	/	/
	Dalgic, et al.	93	31	0	217	31	186	14	0	386	14	0.263	0.608
		CC	TC	TT	C	T	CC	TC	TT	C	T	X^2^	*P*
*TLR6*	Ma, et al. _a	289	47	3	625	53	137	50	7	324	64	0.805	0.370
C745T	Ma, et al. _b	61	88	31	210	150	38	46	26	122	98	2.594	0.107
(rs5743810)	Ma, et al. _c	291	74	10	656	94	78	31	5	187	41	0.696	0.404
	Salvaraj, et al.	0	2	197	2	396	0	3	199	3	401	0.011	0.915

HWE: Hardy-Weinberg Equilibrium.

### Quantitative Data Synthesis

#### TLR1 G1805T polymorphism

Six case-control studies (1684 cases and 1216 controls) on the relationship between the G1805A polymorphisms and the risk of TB were included in the meta-analysis.

**Figure 3:**
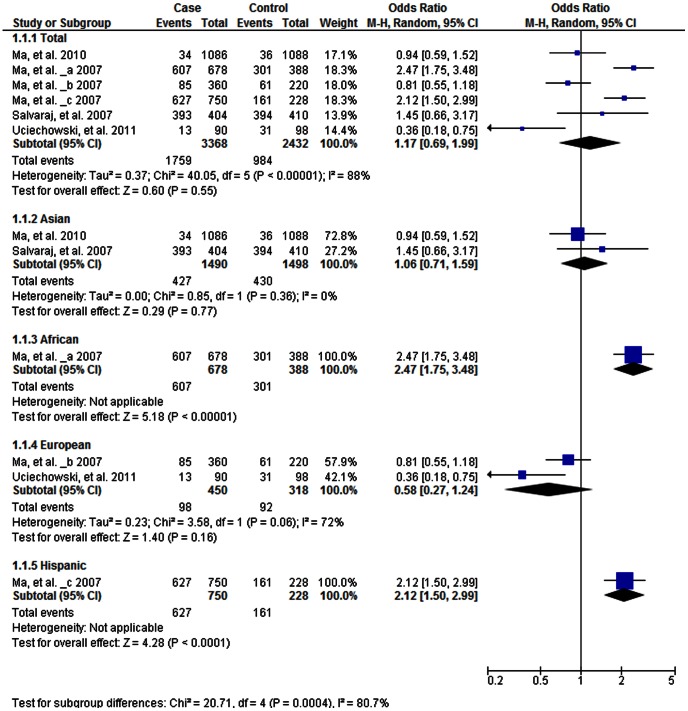
Forrest plot of the association between *TLR1* C1805T and TB risk in allele comparison (T vs. C). Subgroup analysis was performed by ethnicity. OR: odds ratio; CI: confidence interval; df: degrees of freedom.

As shown in Table 3, the heterogeneity of T vs. G for all the studies was analyzed. The χ^2^ value was 40.05, with 5 degrees of freedom (df) and *P*<0.001 in a random effect model. *I* ^2^, another index of the test of heterogeneity, was 88%, suggesting a high heterogeneity. We therefore chose the random-effect model to synthesize the data. The overall OR for the T vs. G alleles was 1.17 (*P = *0.55; Figure 3). No association was found in the allelic frequency with TB risk. The publication bias by Begg’s test showed no significant bias (*P*>0.05; Figure S1). We also performed comparison for the other four genetic models and no associations were found in any of these (Table 3).

**Table 3:** Summary of different comparative meta-analysis results.

Polymorphism	Genetic model	Participants	OR [95%CI]	Z	*P* value	I^2^ %	*P*_het_	Effect model	Begg’s test p>|z|	Egger’s test p>|t|
*TLR1* G1805T	TT+GT vs. GG	2900	1.13 [0.54, 2.36]	0.33	0.74	80	0.0001	R	0.707	0.899
	TT vs. GT+GG	2900	1.43 [0.83, 2.48]	1.29	0.20	68	0.007	R	0.260	0.147
	TG vs. GG	1756	0.94 [0.51, 1.76]	0.18	0.86	68	0.008	R	1.000	0.661
	TT vs. GG	2445	1.31 [0.44, 3.92]	0.48	0.63	77	0.0006	R	1.000	0.670
	T vs. G	2900	1.17 [0.69, 1.99]	0.60	0.55	88	<0.001	R	0.260	0.222
*TLR2* T597C	CC+CT vs. TT	3754	0.90 [0.68, 1.18]	0.77	0.44	69	0.004	R	0.764	0.555
	CC vs. CT+TT	3754	1.12 [0.93, 1.34]	1.20	0.23	42	0.11	F	0.548	0.717
	CT vs. TT	3108	0.87 [0.66, 1.15]	0.99	0.32	67	0.006	R	1.000	0.736
	CC vs. TT	1966	1.00 [0.69, 1.44]	0.01	0.99	63	0.01	R	0.764	0.646
	C vs. T	3754	0.98 [0.83, 1.15]	0.24	0.81	60	0.02	R	0.764	0.689
*TLR2* T1350C	CC+CT vs. TT	2688	1.06 [0.86, 1.31]	0.55	0.58	27	0.24	F	0.133	0.024
	CC vs. CT+TT	2688	1.01 [0.56, 1.79]	0.02	0.98	0	0.52	F	0.707	0.063
	CT vs. TT	2639	1.05 [0.84, 1.30]	0.41	0.68	4	0.39	F	0.133	0.071
	CC vs. TT	2214	1.01 [0.56, 1.82]	0.04	0.97	0	0.52	F	0.452	0.051
	C vs. T	2688	1.05 [0.87, 1.27]	0.54	0.59	43	0.12	F	0.024	0.009
*TLR2* G2258A	AA+AG vs. GG	5077	1.72 [0.89, 3.31]	1.62	0.10	61	0.006	R	0.721	0.041
	AA vs. AG+GG	5077	5.82 [1.30, 26.16]	2.30	0.02	/	/	R	/	/
	AG vs. GG	5061	1.55 [0.78, 3.09]	1.25	0.21	63	0.004	R	1.000	0.041
	AA vs. GG	4877	6.04 [1.34, 27.18]	2.34	0.02	/	/	R	/	/
	A vs. G	5077	1.78 [0.99, 3.20]	1.92	0.05	56	0.01	R	0.592	0.052
*TLR6* C745T	TT+CT vs. CC	1713	0.64 [0.38, 1.06]	1.73	0.08	72	0.03	R	0.296	0.019
	TT vs. CT+CC	1713	0.61 [0.39, 0.97]	2.10	0.04	0	0.41	F	1.000	0.890
	CT vs. CC	1235	0.69 [0.40, 1.19]	1.34	0.18	74	0.02	R	0.296	0.056
	TT vs. CC	1372	0.57 [0.34, 0.95]	2.16	0.03	29	0.24	F	0.296	0.261
	T vs. C	1713	0.66 [0.44, 0.99]	2.01	0.04	65	0.04	R	1.000	0.811

*P*_het_ = *P* value for heterogeneity; OR = odds ratio; CI = confidence interval; F = fixed-effect model; R = random-effect model.

We also performed a stratified subgroup allelic analysis by ethnicity and different results were found in different ethnic populations. Slight decreased risk among Europeans (OR 0.58 [0.27, 1.24], *P* = 0.16; Figure 3) and slight increased risk among Asians (OR 1.06 [0.71, 1.59], *P = *0.77; Figure 3) were observed. However, one study in Africans (OR 2.47 [1.75, 3.48], *P*<0.01; Figure 3) and one in Hispanics (OR 2.12 [1.50, 2.99], *P*<0.01; Figure 3) showed a significant increased risk associated with the 1805T allele.

#### TLR2 T597C polymorphism

Seven case-control studies (2014 cases and 1740 controls) were included in the meta-analysis on the relationship between the T597C polymorphism and the risk of TB.

As shown in Table 3, the heterogeneity of C vs. T for all the studies was analyzed. The χ^2^ value was 15.14, with 6 df and *P* = 0.02 using the random-effect model, while *I* ^2^ was 60, suggesting a modest heterogeneity. We therefore chose the random-effect model to synthesize the data. The overall OR for the C vs. T alleles was 0.98 (95% CI 0.83–1.15) and the test for overall effect, Z value, was 0.24 (*P* = 0.81), indicating no association between this polymorphism and TB. The publication bias by Begg’s test showed no significant publication bias (*P*>0.05). Comparisons for four other genetic models were also performed, and no associations were found either (Table 3).

#### TLR2 T1350C polymorphism

Six case-control studies (1379 cases and 1309 controls) were included in the meta-analysis to determine the relationship between the T1350C polymorphism and the risk of TB.

As shown in Table 3, the heterogeneity of C vs. T for all the studies was analyzed. The χ^2^ value was 8.79, with 5 df and *P* = 0.12 using the fixed-effect model, while *I* ^2^ was 43%, suggesting limited heterogeneity (Table 3). The overall OR for the C vs. T alleles was 1.05 (95% CI 0.87–1.27), and the test for overall effect, Z value, was 0.54 (*P* = 0.59). Comparisons for four other genetic models were also performed, and no associations were found (Table 3). The publication bias by Begg’s test showed no bias in all the genetic models except the allelic model (*P*<0.05), indicating that there might be some publication bias in these studies.

#### TLR2 G2258A polymorphism

Eleven case-control studies (2823 cases and 2254 controls) on the relationship between the *TLR2* G2258A were included in the meta-analysis.

**Figure 4:**
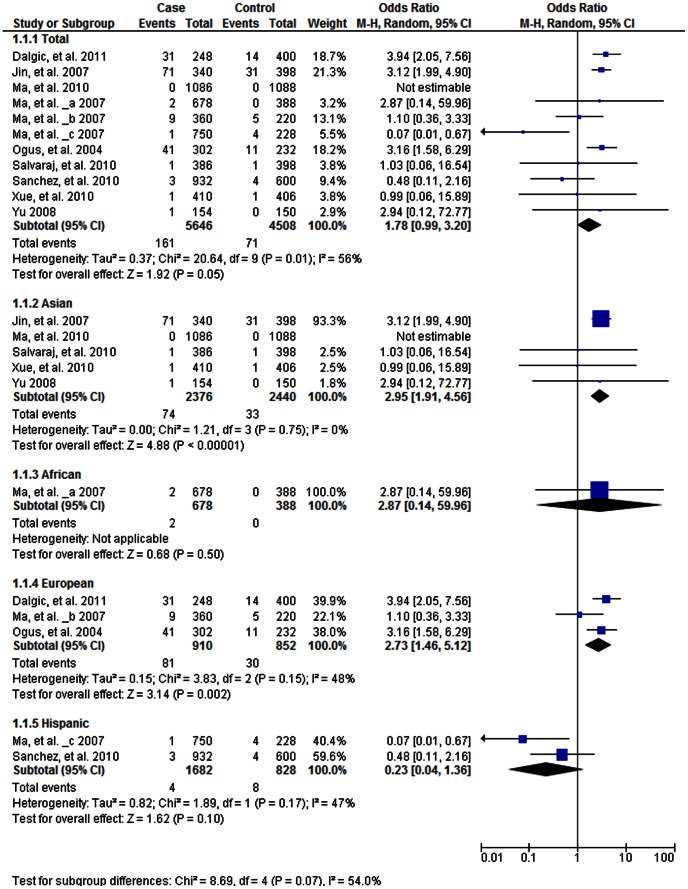
Forrest plot of the association between *TLR2* G2258A and TB risk in allele comparison (A vs. **G).** **** Subgroup analysis was performed by ethnicity. OR: odds ratio; CI: confidence interval; df: degrees of freedom.

As shown in Table 3, the heterogeneity of A vs. G for all the studies was analyzed. The χ^2^ value was 20.64, with 9 df and *P = *0.01 in a random-effect model and *I* ^2^ was 56%, suggesting moderate heterogeneity. The overall OR for the A vs. G alleles was 1.78 (95% CI 0.99–3.20) and the test for overall effect, Z value, was 1.92. (*P* = 0.05; Figure 4). The results suggest an increased risk in the A alleles. In recessive model analysis, the overall OR for the AA vs. AG+GG was 5.82 (95% CI 1.30–26.16, *P* = 0.02) using the random effect model, indicating an association of the AA genotype with TB risk. The Begg’s test did not show publication bias in the studies (*P*>0.05, Figure S2).

We also performed a stratified analysis by ethnicity for the allelic model (A vs. G). Significantly increased risks were found among Asians (OR 2.95, 95%CI 1.91–4.56, *P*<0.001; Figure 4) and Europeans (OR 2.73, 95% 1.46–5.12, *P = *0.002; Figure 4) and decreased risk was found in Hispanic population (OR 0.23, 95% 0.04–1.36, *P* = 0.10; Figure 4), while there was a slight but not significant increased risk for A allele in Africans (P = 0.50; Figure 4).

#### TLR6 C745T polymorphism

Four case-control studies (1093 cases and 620 controls) were included in the meta-analysis on the relationship between the *TLR6* C745T polymorphism and the risk of TB.

**Figure 5:**
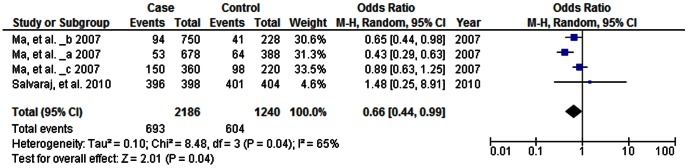
Forrest plot of the association between *TLR6* 745T and TB risk in allele comparison (T vs. C). OR: odds ratio; CI: confidence interval; df: degrees of freedom.

As shown in Table 3, the heterogeneity of T vs. C for all the studies was analyzed. The χ^2^ value was 12.64, with 3 df and *P* = 0.005 using the random-effect model, while *I* ^2^ was 76, suggesting a high heterogeneity. The overall OR for the C vs. T alleles was 0.66 (95% CI 0.44–0.99) and the test for overall effect, Z value, was 1.34 (*P* = 0.04; Figure 5), indicating a protective association between the T allele and TB. In addition, in recessive model analysis, the overall OR for the TT vs. CT+CC was 0.61 (95% CI 0.39–0.97, *P* = 0.04), also indicating a decreased TB risk in subject with the TT genotype. The publication bias by Begg’s test showed no significant publication bias (*P*>0.05; Figure S3).

## Discussion

In this study, we performed a meta-analysis to assess the association between five extensively studied polymorphisms (*TLR1* G1805T, *TLR2* T597C, T1350C, G2258A and *TLR6* C745T) and TB risk reported until 1^st^ January 2013. The meta-analysis revealed an association between the *TLR2* 2258AA genotype and *TLR6* 745TT and TB risk. At the same time, different genetic models and ethnicity subgroups comparisons were also performed in *TLR1* G1805T, *TLR2* G2258A polymorphisms. *TLR1* 1805T allele was associated with TB in Africans and American Hispanics, and *TLR2* 2258A allele was associated with TB in Asians and Europeans. However, the *TLR2* T597C, *TLR2* T1350C polymorphism did not show significant association with TB.

**Figure 6:**
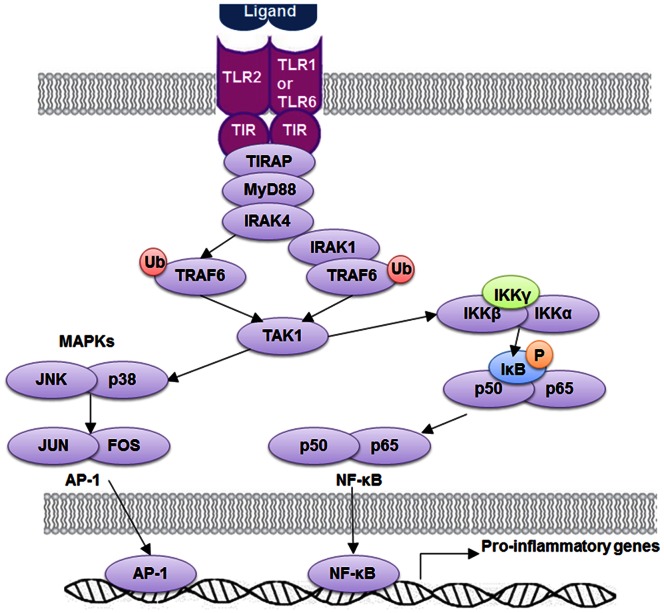
Signaling pathway of TLR-1, -2 and -6. TLR2 ligands induce conformational changes of TLRs that allow interactions of TLR2 and TLR-1, -6. This heterodimer could recruit adaptor proteins MyD88 and TIRAP. MyD88 can activate NF-κB and AP-1 through IRAKs, TRAF6, TAK1, and IKK complex, resulting in induction of pro-inflammatory genes in macrophages and cDCs. TLR: Toll-Like Receptor; TIR: Toll-IL1-Receptor Domain; TIRAP: TIR domain containing adaptor protein; MyD88: Myeloid Differentiation Primary Response Gene 88; IRAK: Interleukin-1 Receptor associated kinase 1; TRAF: TNF Receptor Associated Factor; TAK: TGF-β-Actiated Kinase; IκB: Inhibitor of NF-κB; IKK: IκB kinase.

TLR-1, -2, and -6 are critical components in Mtb recognition and signaling [34]–[36]. As shown in Figure 6, ligand recognition leads to TLR2 dimerization with TLR1 or TLR6 that brings together their TIR domains and triggers TLR2 tyrosine phosphorylation. This would serve as docking platforms to enable recruitment of MyD88 [35]. MyD88 interacts with TIRAP via TIR-TIR domain interactions, serving as a scaffold to recruit IL-1 receptor associated kinase (IRAK) 4 and IRAK1. Clustered IRAKs could undergo auto-phosphorylation and kinase activation. Phosphorylated IRAK4 would recruit TNF receptor associated factor 6 (TRAF6), leading to their ubiquitination. Ubiquitinated TRAF6 recruits TGF-β-activated kinase (TAK) 1 by engaging ubiquitin recognition domains within inhibitor of NF-κB (IκB) kinase (IKK)-γ and TAK-interacting proteins [35]. On the other hand, phosphorylated IRAK1 could also undergoes ubiquitination via recruitment of pellinos and TRAF6, resulting in direct recruitment of IKK-γ [35]. Finally, these processes activate TAK1 and the IKK complex and place them into close proximity, promoting TAK-mediating activation of MAPK and IKK-β, resulting in nuclear translocation of transcription factors, such as AP-1 and NF-κB, that induces transcription of inflammatory genes [34]. Because of their essential roles in Mtb recognition process, polymorphisms in TLR-1, -2 and -6 are hypothesized to be associated with susceptibility to TB and other infectious diseases.

The G2258A polymorphism in TLR2 was first identified by mutation screen assay in healthy study subjects, which cause the 753 amino acid substitution from Arg to Gln in the conserved TIR domain (Figure 1) [37]. In 293T cells transfected with wild-type or G2258A TLR2 constructs, 2258A allele has a significant decreased NF-κB response against bacterial peptides from *B. burgdorferi*, *T. pallidum* and *R. akari* in comparison to the wild-type, resulting in an attenuated immune response [37], [38]. Recently, another study looked into the mechanisms behind this and demonstrated that G2258A rendered TLR2 signaling-deficient by impairing TLR2 tyrosine phosphorylation, TLR2-TLR6 hetero-dimerization, and recruitment of TIRAP and MyD88 in transfected HEK293 cells, without affecting TLR2 expression [39]. This study also showed that polymorphism in the TIR domain could change the electrostatic potential of the DD loop and α-D region, which interact with the BB loops of TLR1 and TLR6, and thus impair their dimerization. Because of the functional importance of this SNP, numerous studies have investigated the association of this polymorphism with TB, urinary tract infection and other infectious diseases [25], [40], [41]. One of the studies found that atopic dermatitis (AD) patients carrying the 2258A allele showed higher levels of total serum IgE as well as of superantigen-specific IgE and had a higher risk of getting the disease [42]. Another study reported that G2258A TLR2 allele in transfected HEK293 cells had a decreased IL-8 secretion after stimulation by lipoteichoic acid, heat-inactivated Staphylococcus aureus or triacylated lipopeptides, and it was associated with suppressed IL-8 production by monocytes in AD patients as well [43]. Therefore, this SNP might have a significant functional impact on the TLR2 signaling. In our analysis, *TLR2* G2258A was linked to TB in the recessive model (AA vs. AG+GG), but other polymorphisms in the *TLR2* did not associate with TB. The subgroup analysis by ethnicity showed that G2258A was associated with an increased risk of TB in Asian and European populations. The reasons for this difference of association in different populations were unclear, but the differences in the frequencies of the polymorphisms among different races might contribute to this heterogeneity. In addition, numerous polymorphisms outside the *TLR2* gene can contribute to the host susceptibility to TB. These polymorphisms might differ greatly in various ethnic groups with different evolutionary backgrounds. These differences, along with gene-gene interactions and environmental and cultural factors, and even the variations in Mycobacterium strains, made understanding the observed differences between ethnic groups even more complicated.

*TLR1* T1805G is a common non-synonymous polymorphisms located in the trans-membrane domain of TLR1 molecule (Figure 1), causing a substitution of isoleucine with serine. Studies showed that this substitution affected the TLR1 surface trafficking and led to an impairment of the inflammation pathway, and it might be the most common SNP affecting TLR function identified in any population [44]. *TLR1* T1805G transfected HEK293 cells had substantially greater basal and lipopeptide-induced or Mtb extracts-induced NF-κB signaling [12]. Furthermore, individuals with the 1805TT genotypes produced substantially more IL-6 than those with the 1805GG in a lipopeptide-stimulated whole-blood cytokine assay [12]. In another study, TLR1 1805GG carriers have an impaired cell surface expression of TLR1 and impaired inflammatory response to lipopeptide agonists for TLR1/2 [44]. It was further validated that this effect was caused by T1805G, not a genetically linked allele, by transfection assay [44]. This study also discovered that 1805G was a common SNP and was associated with an increased risk of leprosy [44]. Different groups using different methods have also replicated these findings in various models [45], [46]. In a subgroup analysis of TLR polymorphisms and susceptibility to inflammatory bowel disease, SNP T1805G was associated with Crohn’s disease that was confined to the ileum [47]. These observations together with association studies in other infectious diseases [44], [48] suggested that *TLR1* T1805G could potentially impact the innate immune response and clinical susceptibility to TB, which is consistent with our meta-analysis results that 1805G was associated with TB susceptibility in Africans and American Hispanics. The reason why different results were observed in different populations might be that the frequency of this SNP varied widely across different populations [12].

In our analysis, the *TLR6* 745TT genotype was associated with a reduced TB risk. Given the location of this non-synonymous SNP in the extracellular domain (Figure 1), people have speculated that this SNP might alter ligand recognition. One study showed that this SNP was associated with altered IL-6 secretion from the whole blood samples of the healthy people in response to di-acylated lipopeptide, Mtb lysate and Bacille Calmette-Guerin (BCG) [13]. In addition, this SNP allele was associated with different NF-κB signaling in response to di-acylated lipopeptide, PAM2 or Mtb in an HEK293 cell line reconstitution assay [13]. Another study re-stimulated the whole blood drawn from the newborn treated with BCG and found that individuals with 745T alleles had significantly higher levels of INF-γ, IL-2, and IL-13 [49]. The PBMCs from these individuals also secreted higher amounts of IL-6 and IL-10 after stimulation with lipopeptides, Mtb lysate and BCG [49]. Because inability to produce or respond to IFN-γ in host has been associated with higher susceptibility to disseminated mycobacterial infection [50], the 745C allele that was linked to lower level of IFN-γ response might thus increase TB risk. Therefore, the 745T allele, which is associated with an elevated IFN-γ response against Mtb [49], may confer protection against TB. This is consistent with the results in our meta-analysis.

However, there are still some limitations in our meta-analysis that should be considered when explaining the present results. One of our limitations is the limited number of studies included in this review. Our review reported an interesting preliminary conclusion, but this must be validated by future large-scale and functional studies in different populations. Another limitation is that we mainly focused on the most studied coding variants of candidate genes TLR-1, -2 and -6 and their association with TB risk and excluded the effects of other genetic variants. Other polymorphisms such as *TLR2* Arg677Trp polymorphism [51] microsatellite polymorphisms in *TLR2* gene might also linked to TB and have effects on our candidate SNPs. [52]. These polymorphisms were not included in this systematic review because of insufficient studies available in the database. In addition, the polymorphisms besides the included ones or even the ones in other genes could also affect our results. Therefore, genome-wide association studies (GWAS) in different ethnic groups are needed to validate our results. To date, four GWAS have been performed to study TB susceptibility [53]–[56]. These studies identified polymorphisms on chromosomes 18q11.2 and 11q13 in African but no polymorphism in Asian to be associated with TB. Meta-analysis of the GWAS study might provide more power to detect SNP linked to complex diseases [57]. Therefore the meta-analysis of these studies and future studies in various populations might provide more information on TB risk loci and polymorphism-polymorphism interactions.

Understanding the relation among the TLR polymorphisms, innate immunity and TB susceptibility is also valuable in translational research and personalized medicine. Although susceptibility to TB is historically ascribed to an inadequate immune response that fails to control infecting mycobacteria, people have find that susceptibility to Mycobacterium can result from either inadequate or excessive acute inflammation [4]. Although lack of TLR signaling might promote the TB development [36], excessive acute inflammation induced by TLR2 activation can lead to severe tissue damages [58]. Recently, Tobin and colleagues have investigated the genetic balance that maintains the host between the two extremes of failed immunity and damaging hyper-immunity [4]. By stratifying tuberculous meningitis patients from a historic randomized trial by *ITA4H* genotype, they revealed both different outcomes and different responses to dexamethasone, which might promote the personalized application of eicosanoid-targeting drugs [4]. As drugs targeting TLRs are being developed for infectious and inflammatory diseases including TB [59], host genotype-specific therapy based on knowledge of TLRs polymorphisms may optimize the inflammatory response to Mtb infection in the future.

In conclusion, this systematic review summarized the association between TLR-1, -2, and -6 polymorphisms and TB susceptibility. Our results indicate that *TLR2* G2258A is associated with increased TB risk, especially in Asians and Europeans. *TLR1* G1805T is associated with increased TB in Africans and American Hispanics. *TLR6* C745T is associated with decreased TB risk. Whereas, *TLR2* T597C and *TLR2* T1350C polymorphisms did not show significant association with TB. Our systematic review and meta-analysis reported an interesting preliminary conclusion, but this must be validated by future large-scale studies in different populations. Whether the association between these polymorphisms and the TB risk was due to causal effect need to be further studied by functional studies.

## Supporting Information

Figure S1 **Funnel plot analysis to detect publication bias for association of** ***TLR1*** **C1805T and TB risk in allele comparison (T vs. C).**
(TIF)

Figure S2 **Funnel plot analysis to detect publication bias for association of** ***TLR2*** **G2258A and TB risk in allele comparison (A vs. G).**
(TIF)

Figure S3 **Funnel plot analysis to detect publication bias for association of** ***TLR6*** **C745T and TB risk in allele comparison (T vs. C).**
(TIF)

Table S1 **PRISMA 2009 checklist.**
(DOC)

Table S2 **Exclusion criteria for excluded studies.**
(DOC)
