# Supplementary figures and images for: Toll-Like Receptor -1, -2, and -6 Polymorphisms and Pulmonary Tuberculosis Susceptibility: A Systematic Review and Meta-Analysis
Source: PLoS One. 2013 May 14;8(5):e63357. doi: 10.1371/journal.pone.0063357 (PMC3653945; doi:10.1371/journal.pone.0063357)

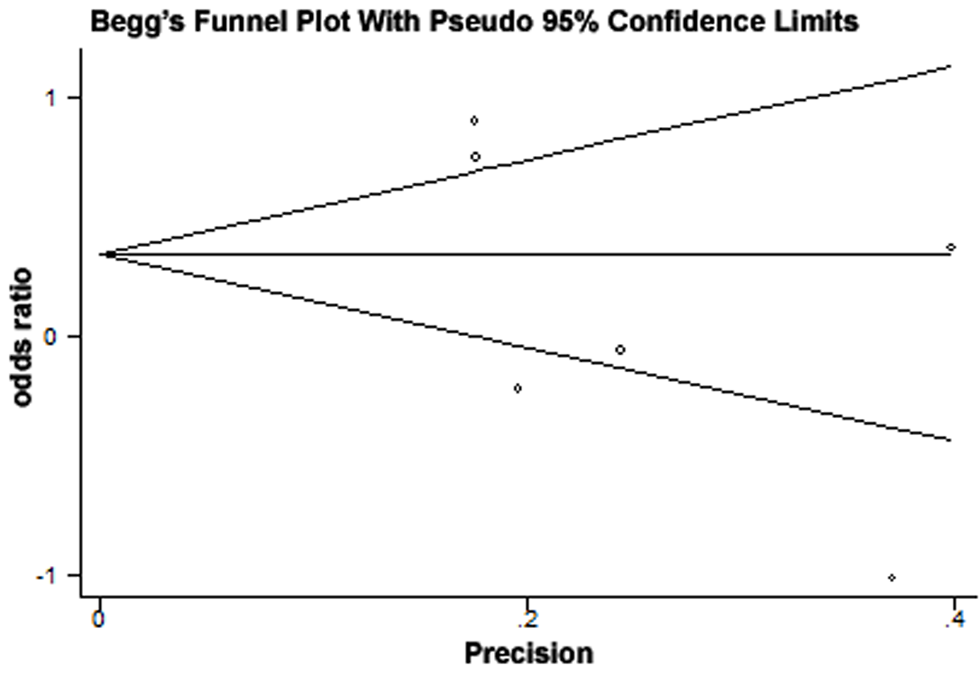

Supplement: Figure S1 — Funnel plot analysis to detect publication bias for association of TLR1 C1805T and TB risk in allele comparison (T vs. C). (TIF) [file pone.0063357.s001.tif]

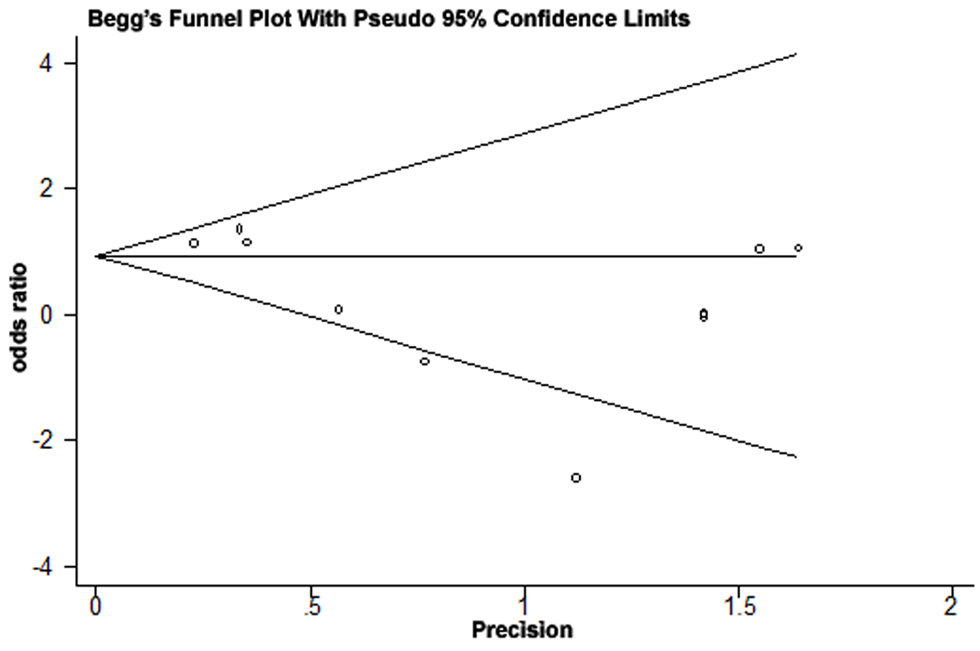

Supplement: Figure S2 — Funnel plot analysis to detect publication bias for association of TLR2 G2258A and TB risk in allele comparison (A vs. G). (TIF) [file pone.0063357.s002.tif]

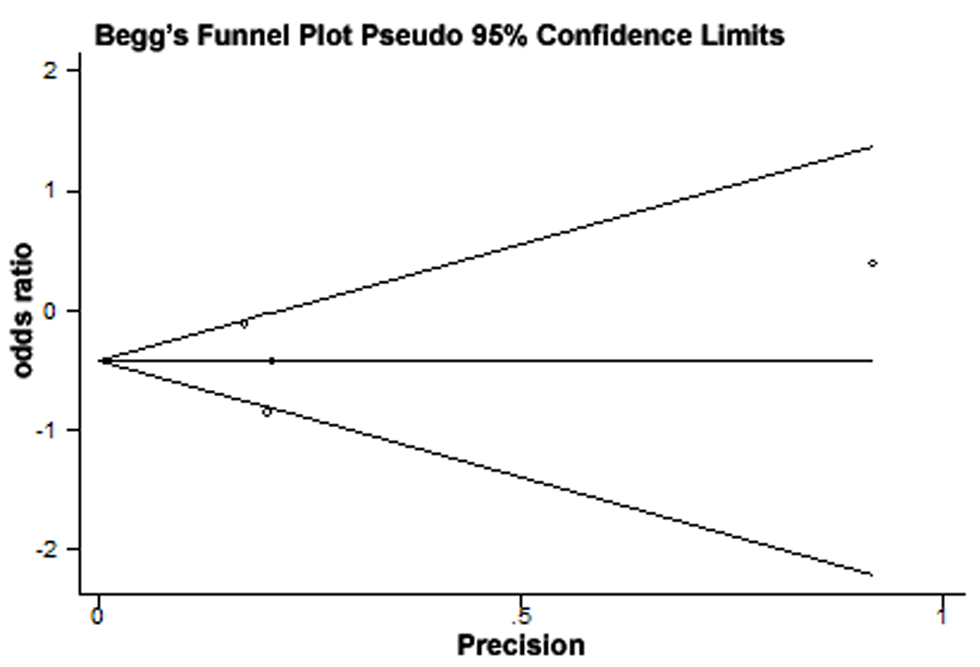

Supplement: Figure S3 — Funnel plot analysis to detect publication bias for association of TLR6 C745T and TB risk in allele comparison (T vs. C). (TIF) [file pone.0063357.s003.tif]
