# Supplementary material for: Toll-Like Receptor -1, -2, and -6 Polymorphisms and Pulmonary Tuberculosis Susceptibility: A Systematic Review and Meta-Analysis
Source: PLoS One. 2013 May 14;8(5):e63357. doi: 10.1371/journal.pone.0063357 (PMC3653945; doi:10.1371/journal.pone.0063357)
Supplement: Table S2 — Exclusion criteria for excluded studies. (DOC) [file pone.0063357.s005.doc]

Table S2. Exclusion criteria for excluded studies.

| **Exclusion criteria** | **Articles** |
| --- | --- |
| Not on the target polymorphisms | 1. Ben-Ali M, Barbouche MR, Bousnina S, Chabbou A, Dellagi K (2004) Toll-like receptor 2 Arg677Trp polymorphism is associated with susceptibility to tuberculosis in Tunisian patients. Clin Diagn Lab Immunol 11: 625-626. 2. Fitness J, Floyd S, Warndorff DK, Sichali L, Malema S, et al. (2004) Large-scale candidate gene study of tuberculosis susceptibility in the Karonga district of northern Malawi. Am J Trop Med Hyg 71: 341-349. 3. Olesen R, Wejse C, Velez DR, Bisseye C, Sodemann M, et al. (2007) DC-SIGN (CD209), pentraxin 3 and vitamin D receptor gene variants associate with pulmonary tuberculosis risk in West Africans. Genes Immun 8: 456-467. 4. Xue Y, Jin L, Li AZ, Wang HJ, Li M, et al. (2010) Microsatellite polymorphisms in intron 2 of the toll-like receptor 2 gene and their association with susceptibility to pulmonary tuberculosis in Han Chinese. Clinical Chemistry & Laboratory Medicine 48: 785-789. 5. Yim JJ, Lee HW, Lee HS, Kim YW, Han SK, et al. (2006) The association between microsatellite polymorphisms in intron II of the human Toll-like receptor 2 gene and tuberculosis among Koreans. Genes and Immunity 7: 150-155. |
| Needed data could not be extracted from the article | 1. Kobayashi K, Yuliwulandari R, Yanai H, Naka I, Lien LT, et al. (2012) Association of TLR polymorphisms with development of tuberculosis in Indonesian females. Tissue Antigens 79: 190-197. 2. Motsinger-Reif AA, Antas PRZ, Oki NO, Levy S, Holland SM, et al. (2010) Polymorphisms in IL-1 beta, vitamin D receptor Fok1, and Toll-like receptor 2 are associated with extrapulmonary tuberculosis. Bmc Medical Genetics 11: 37. 3. Velez DR, Wejse C, Stryjewski ME, Abbate E, Hulme WF, et al. (2010) Variants in toll-like receptors 2 and 9 influence susceptibility to pulmonary tuberculosis in Caucasians, African-Americans, and West Africans. Human Genetics 127: 65-73. |
